# Biosecurity Versus African Swine Fever—Making, Acceptance, and Results of a German Online Assessment Tool

**DOI:** 10.3390/pathogens14060524

**Published:** 2025-05-23

**Authors:** Nicolai Denzin, Nora Wieneke, Maria Gellermann, Carola Sauter-Louis, Barbara Grabkowsky

**Affiliations:** 1 Institute of Epidemiology, Friedrich-Loeffler-Institut, Federal Research Institute for Animal Health, 17493 Greifswald-Insel Riems, Germany; carola.sauter-louis@fli.de; 2 Faculty of Electrical Engineering and Computer Science, Hochschule Stralsund, 18435 Stralsund, Germany; nora.wieneke@hochschule-stralsund.de; 3 Vechta Institute of Sustainability Transformation in Rural Areas (VISTRA), University of Vechta, 49377 Vechta, Germany; maria@holst-gellermann.de (M.G.); barbara.grabkowsky@uni-vechta.de (B.G.)

**Keywords:** African swine fever, biosecurity, online tool, assessment, ASP-Risikoampel, ASF risk traffic light

## Abstract

African swine fever (ASF), a viral hemorrhagic disease with exceptionally high lethality in domestic pigs and Eurasian wild boar, reached Germany in 2020, with the confirmation of the first case in a wild boar next to the border to Poland. Since then, 6621 cases in wild boar but only 19 outbreaks in domestic pigs were confirmed. Biosecurity is crucial in preventing the infection of domestic pig holdings. Already in 2019, an online assessment tool, the so-called “ASP-Risikoampel” (ASF risk traffic light), was launched. It enables farms to identify ASF-specific weaknesses and take targeted measures to minimize risks/optimize the biosecurity standard anonymously and free of charge. The development of the tool incorporating expert opinion elicitation in a Delphi process is detailed and the results of 2290 self-assessments of farms between 2019 and 2023 are evaluated. The proportion of tool utilization relative to the average number of holdings in Germany in this time span was 11.9% with marked differences between the federal states. Most of the farms achieved biosecurity scores above 66.7%, qualifying for a “green traffic light”. The results were significantly different among the federal states. The best performing states were those with the largest mean farm size. The latter was significantly correlated with performance on the farm level.

## 1. Introduction

African swine fever (ASF) is a viral hemorrhagic disease with exceptionally high lethality in domestic pigs and Eurasian wild boar. Despite its limited host range and absent zoonotic potential, its socio-economic impact is very high. In 2007, ASF virus (ASFV), which has its roots in a sylvatic cycle in Sub-Saharan Africa, was introduced into Georgia. Subsequently, the virus spread to the Trans-Caucasian region and reached the Russian Federation, from where the virus moved farther [1]. In 2014, ASF reached the European Union; the first case of ASF in wild boar was confirmed in January in Lithuania, February in the eastern part of Poland, followed by Latvia and Estonia [2]. By 2023, already 14 Member States of the European Union were affected through further spread [3]. In 2019, the first cases of the disease were confirmed in a dead wild boar in western Poland only about 80 km from the border with Germany. The latter case could not be linked epidemiologically to the previously reported cases of ASF in Poland, as it was located 300 km away from the previously affected areas. The virus probably moved into western Poland by human factor rather than as a result of wild boar migration [2]. In a population of infected wild boars, ASF spreads at a rate of 10–12 km per year in the absence of an additional “human” factor, which, among other factors, plays a key role in the advancement of the disease over long distances (up to several hundred kilometers) [4]. Germany had introduced preventive measures, such as the increased monitoring of wild boars and domestic pigs and the construction of a fence along the Polish border to prevent wild boar migration into the adjacent German federal states Brandenburg and Saxony [2]; nonetheless, in September 2020, Germany’s Agriculture Minister confirmed the first case of ASF in a fallen boar found in the Spree-Neiße district of Brandenburg [5]. Since then, 6621 cases in wild boar and 19 outbreaks in domestic pigs (as of 9 January 2025) were confirmed in Germany [6]. Until July 2024, all cases and outbreaks, except two single outbreaks in the federal states Lower Saxony and Baden-Wuerttemberg attributed to human factors, were confined to the eastern federal states Brandenburg, Saxony, and Mecklenburg–Western Pomerania, all bordering Poland. From July to August 2024, cases in wild boar were detected for the first time also in the western federal states Hesse, Rhineland Palatinate, and Baden-Wuerttemberg, followed by the first outbreaks in domestic pigs in Hesse and Rhineland Palatinate. The current ASF panzootic is characterized by self-sustaining cycles of infection in the wild boar population [7], which constitutes a newly identified component involving virus transmission by wild boar and virus survival in the environment, the so-called wild boar–habitat cycle, which is characterized by both direct transmission between infected and susceptible wild boar and indirect transmission through carcasses in the habitat [8]. Spill-over and spill-back events occur from wild boar to domestic pigs and vice versa [7].

In the absence of a vaccine, biosecurity is key in preventing the introduction and spread of ASF [1]. Countries free from a disease try to maintain their status through trade restrictions, demands for health certification for imports, and border controls. These measures might fail but ensuring this kind of territorial biosecurity is especially difficult if the disease is vector-borne, particularly if flying insects are involved, or is spread by wildlife, as in the case of ASF. Farm biosecurity then constitutes the final defense line in order to protect domestic pigs. But, it is a challenging task and depends heavily on the awareness of risk factors and legal requirements followed by everyday compliance with the identified procedures for risk reduction.

In order to enhance awareness and implementation of biosecurity measures, an online tool for an anonymous self-assessment of the biosecurity standard was developed for domestic pig farmers, the so-called ASF traffic light (“ASP-Risikoampel”). The latter was an adaptation of the AI (avian influenza) traffic light launched already in 2019 [9]. This paper details the development of the new ASF tool, acceptance by farmers, and results of the biosecurity assessments.

## 2. Materials and Methods

Based on a literature search and expert opinion, relevant risk factors for ASFV introduction into pig farms were identified. Major sources of information were the biosecurity measures laid down in the German directive on the hygienic requirements for pig holdings (Swine Husbandry Hygiene Regulation) [10], the European Animal Health Law [11] and directives delegated thereof, as well as practical guidelines, handbooks, and recommendations from governmental, agricultural, and industry contexts. The identified risk factors were then addressed by questions suitable to retrieve information on the farm status with respect to these factors. Next, two to three pre-defined answers were attributed to each question. This led to a multiple-choice system of questions and answers, which helped to obtain comparable results from all participating farms. The predefined answers were coded according to the level of compliance with the respective biosecurity aspect they related to. The first answer refers to complete compliance with the possible biosecurity measures (coded as 1) with respect to the specific risk factor (question), and the second answer refers to the absence of the respective measures (coded as 0). For some questions, an additional intermediate level of compliance (coded as 0.5) was provided as a possible answer. If relevant, the answer “not applicable” (to the assessed farm) was also added as an option (e.g., cleansing and disinfection of the feed storage; if there was no grain storage on the farm, “not applicable” could be selected). Answers were regarded as mutually exclusive, i.e., only one option could be marked. Questions specified as “not applicable” were excluded from the calculation of the risk estimate (see below).

Risk factors, questions, and the respective answer options were reviewed and complemented by an expert panel of 24 experts from veterinary authorities (n = 8), veterinary research institutions (n = 6), veterinary practice (n = 2), pig producers (n = 1), laboratories (n = 1), as well as relevant national and regional associations of veterinarians, farmers, and producers (n = 6) with respect to comprehensiveness, intelligibility, and plausibility.

The expert panel was asked to participate in a two-stage Delphi study to derive specific weights for each risk factor for ASFV introduction. The objective was to reach a consensus among the experts, and the degree of consolidation of expert opinion was assessed (see below). The experts received a compilation of the questions and answers used in the questionnaire by e-mail. They were asked to first allocate a weight score of 1, 2, or 3 (reflecting increasing importance) to each question and then have a second look at those questions they had assigned a weight of 3 to. If the experts believed that the underlying risk factor of a question was of extraordinary importance, they were asked to assign a weight of 5. The weights assigned by the experts in this first poll (1st Delphi round) were analyzed and the minimum, maximum, mean, and median were calculated. These measures were communicated to the experts, who were then asked to re-evaluate their individual estimates (2nd Delphi round) in light of the summarized panel rating.

To assess the impact of the 2nd Delphi round on the spread of scores assigned to the individual risk factors, coefficients of variation [12] were calculated for the results of the 1st and 2nd Delphi rounds and the differences expressed as percentages of change.

The maximum achievable biosecurity score was calculated as the sum of the products maximum answer code (1.0) times the risk factor (question) weight for all applicable questions. The biosecurity level of the farm was then expressed as the percentage of the achieved score relative to the maximum score. In addition, it was displayed using a traffic light color code: <33.3%, high risk, red; 33.3–66.7%, intermediate risk, yellow; and >66.7%, low risk, green. A to-do list with recommendations for improving biosecurity, ranked top-down according to the risk factor relevance (product as specified above), for the specific farm was also provided.

The online tool was programmed on a Stud.IP platform. The “Online course support for classroom teaching” (Stud.IP) [13] is an internet-based, open-source platform to support teaching and research [14]. Stud.IP has a modular structure and may be expanded and upgraded via standardized extensions, so-called plugins.

The use of the online tool was anonymous, i.e., an assignment of the results to users via an IP or other data means was not possible during or after use. For an evaluation on a regional basis, five assignment parameters, i.e., country, federal state, production type (fattening, breeding, or mixed), farm size (heads of pigs), and the background of use [primary/secondary (for the same farm) professional use; academic interest, only] were queried.

The data themselves were stored on a server at the IT center of the University of Vechta and may only be used for an evaluation after consultation with the project team.

Participation was without any (particularly without official) consequences for the user. The online tool only serves as an aid for an evaluation of individual farms under the responsibility of the farmers themselves. The user is provided with the option to download (as a PDF document), print, or save the generated recommendations for biosecurity optimization and the to-do (check) list. Access to and use of the online tool are free of charge.

The acceptance of the online tool was evaluated for the years 2019 to 2023. For this purpose, the number of times the tool was used was analyzed and a distinction made as to whether these were primary or secondary professional uses. To evaluate potential differences in the acceptance with respect to the 16 German federal states, the respective proportion of utilization was calculated. The number of uses per federal state was standardized based on the average number of farms reported for the years 2019 to 2023 in the respective states [15]. In order to examine the possible influence of the geographical distance to cases of African swine fever in Germany on the willingness to use the online tool, the user’s distance to the epidemic was determined. As, for data protection reasons, only the user’s federal state was known as most precise information on the geographical location of the farm, the distance between the geographical centroid of the respective federal state and the centroid of cases of ASF in feral pigs in Germany as of 12 December 2023 (5598 cases, first case on 10 September 2020) was used as the distance. Linear regression was employed to assess the correlation between proportion of utilization and distance. The so-called city states (Berlin, Hamburg, and Bremen) with no registered pig holdings were excluded from the analysis.

The distributions of overall scores, main category scores, and subcategory scores of the users between 2019 and 2023 were illustrated as box-and-whisker plots in the style of Tukey [16].

In order to evaluate the effect of the farm size on the performance of farms in the assessment, the achieved overall scores of the farms were regressed (linear regression) on the farm size class indicated by participating farmers (drop-down menu from 100 to 1000 heads of pigs in steps of 100, from 1000 to 10,000 in steps of 1000).

Data analysis and visualization were carried out using R Statistical Software (The R Project for Statistical Computing, Vienna, Austria, version 4.3.1) [17]. Maps were created employing the open-source software Karten-Explorer (Friedrich-Loeffler-Institut, Greifswald, Germany, version 2.2.1) [18].

## 3. Results

### 3.1. Development of the Online Assessment Tool

**Figure 1:**
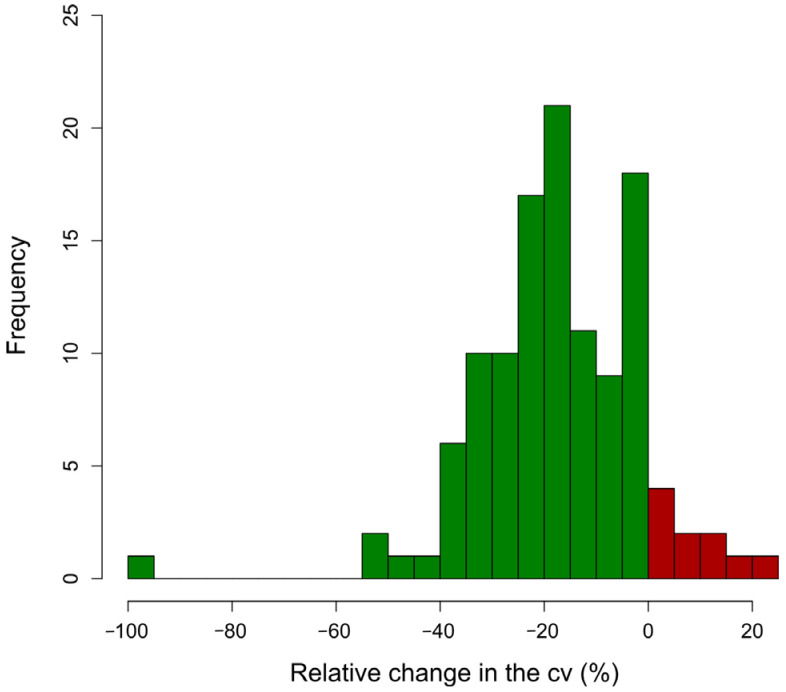
Histogram of the relative change in the coefficient of variation (cv) of the weights allocated to the 117 questions by the expert panel from the 1st to the 2nd Delphi rounds.

**Figure 2:**
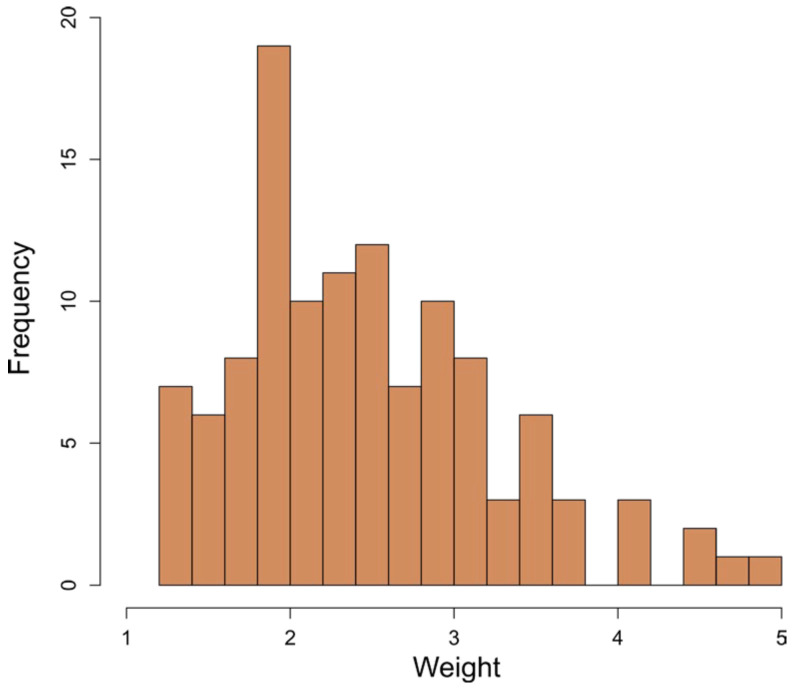
Histogram of the final average weights allocated to the risk questions by the 24 experts.

Altogether, 117 relevant questions (Table S1 with a glossary in Table S3) were identified and confirmed by the expert panel, and were grouped in three main categories: farm features, compound/stable interface, workflow and farm management, referred to as management below. In the Delphi process, the consistency of the weights allocated by the experts to the questions was improved by 17.0% on average (mean coefficient of variation after the 1st and 2nd Delphi rounds, respectively: 38.3%/31.8%). For 107 questions, the consistency was improved, with a maximum of 100%; for 10 questions, the variation increased, with a maximum of 21.1%. The distribution of changes is illustrated in Figure 1. The final average weights after the 2nd Delphi round ranged from 1.2 to 5.0, with a mean of 2.49 and a median of 2.35. The distribution of average weights is shown in Figure 2.

### 3.2. Acceptance of the Online Assessment Tool

**Figure 3:**
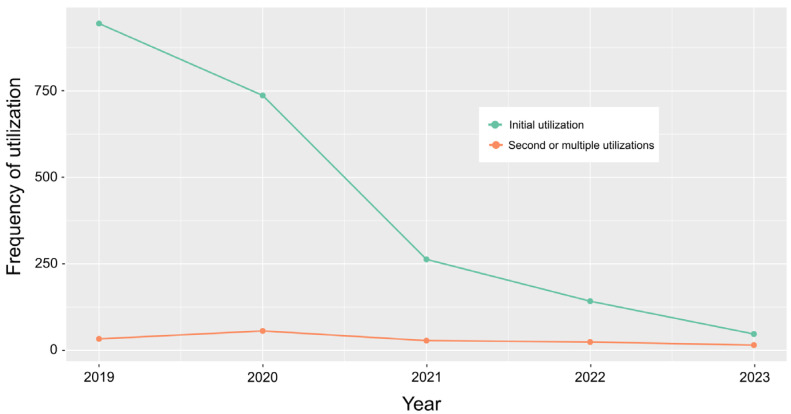
Frequency and intention of utilization from 2019 to 2023.

Between 2019 and 2023, the online tool was used 3423 times. Datasets from test utilizations without relevance for real pig holdings (can be indicated by the user), utilizations from outside Germany, and those without an indication of the federal state or farm size were excluded, leaving 2290 complete datasets (Table S2 with a glossary in Table S3) for analysis, which refers to the proportion of tool utilization relative to the average number of pig holdings in Germany in this time span of 11.9%. The number of initial utilizations and second or multiple utilizations (to be indicated by the user) was 2134 and 156, respectively. The number of initial utilizations was highest (942) in 2019 and declined continuously until 2023; the number of secondary and multiple utilizations fluctuated around 30 over time (Figure 3). Because of the very small number of indicated second and multiple utilizations and the impossibility to assess if the respective initial utilizations were included in the complete data set, the data were analyzed across all types of utilization.

**Figure 4:**
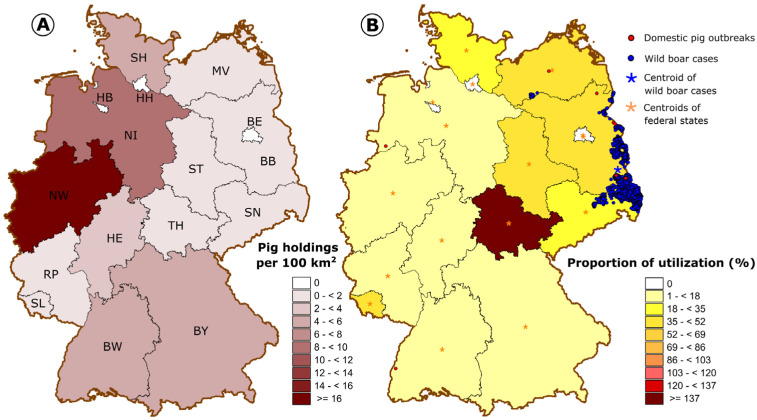
(**A**) Average pig holding density and (**B**) number of tool utilizations relative to the average number of pig farms in the German federal states from 2019 to 2023. SH—Schleswig Holstein, HH—Hamburg, HB—Bremen, SL—Saarland, NI—Lower Saxony, BY—Bavaria, ST—Saxony-Anhalt, BE—Berlin, BB—Brandenburg, SN—Saxony, TH—Thuringia, HE—Hesse, RP—Rhineland Palatinate, BW—Baden-Württemberg, NW—North Rhine-Westphalia, MV—Mecklenburg Western Pomerania.

**Figure 5:**
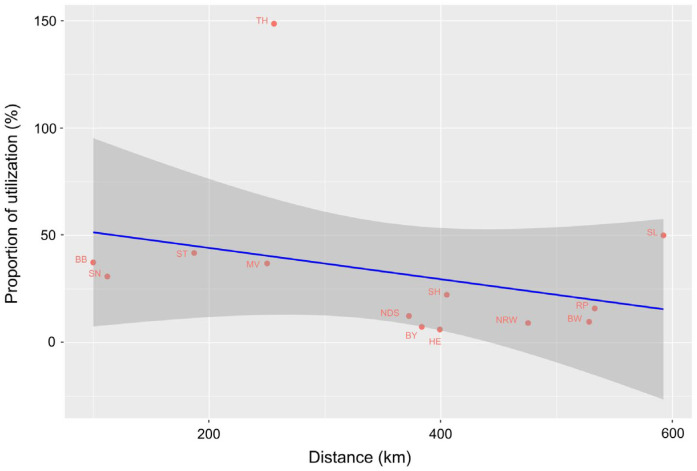
Proportion of tool utilization from 2019 to 2023 in the German federal states regressed on the distance to ASF cases in wild boar with 95% confidence interval. For the federal state abbreviations, see Figure 4. City states without pig holdings (Berlin, Hamburg, and Bremen) were excluded from the analysis.

Pig holding densities are in general higher in the western federal states of Germany (Figure 4A), with a maximum of above 18.2 holdings per 100 km^2^ in North Rhine-Westphalia. The proportion of tool utilizations in the federal states, ranging from 6.2% (Hesse) to 148.6% (Thuringia), was higher in the eastern federal states, which are closer to the wild boar cases, with the exception of the federal state Saarland (50.0%) in the southwest of Germany (Figure 4B). There was a slight negative linear trend for the proportion of utilization regressed on the distance between the centroid of the federal state and the centroid of the ASF cases in wild boar (Figure 5), which was not statistically significant (*p* = 0.30).

### 3.3. Assessment of the Biosecurity Scores

**Figure 6:**
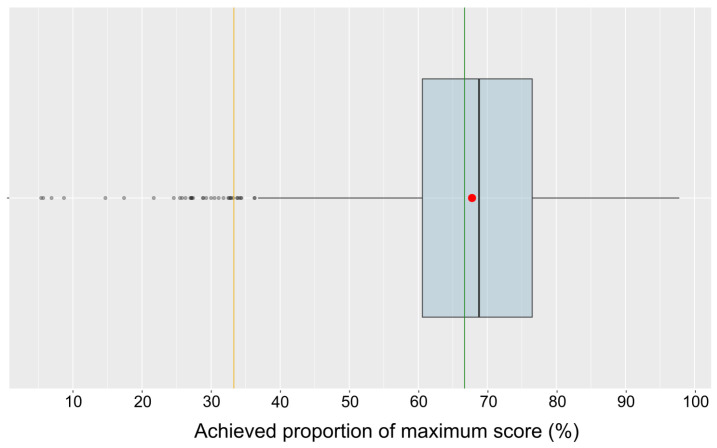
Box-and-whisker plot of the overall scores in Germany. The mean is indicated as a red dot and the risk class thresholds as lines (orange and green).

The distribution of the overall scores of the pig holdings in the biosecurity assessment of the online tool is depicted in Figure 6. The distribution was skewed to the left, with the mean (67.8%) slightly lower than the median (68.8%). Only 30 (1.3%) of the participating holdings yielded results below 33.3%, i.e., belonged to the high-risk farms, whereas 57.6% of the holdings scored higher than the 66.7% threshold for low-risk farms. For details, see Table 1.

**Figure 7:**
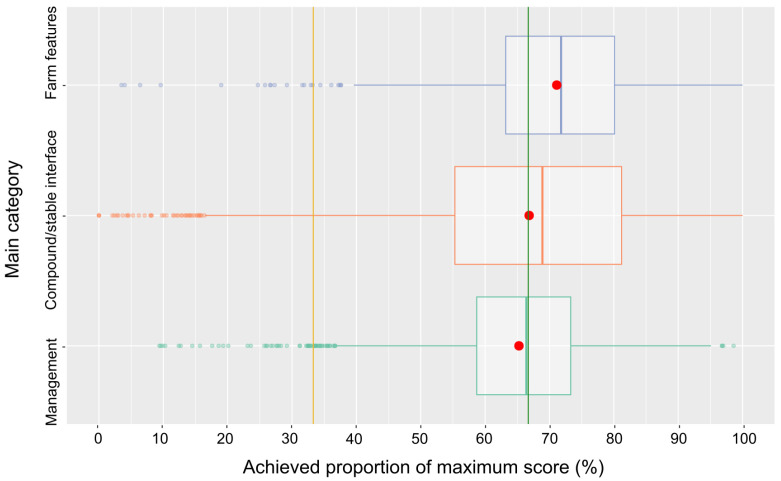
Box-and-whisker plot of the scores in the three main categories. The means are indicated as red dots and the risk class thresholds as lines (orange and green).

The distributions of the scores for the three main categories (Figure 7) were skewed to the left, with medians and means above or close to the 66.7% threshold (low risk). Among the categories, the median of the category management was lowest as compared to the other main categories, but only 1.5% of the scores were in the high-risk class. On the contrary, regarding the compound/stable interface with a median well above the 66.7% threshold, the percentage of scores falling into the high-risk class was highest (7.0%) (for details, see Figure 7 and Table 1).

**Figure 8:**
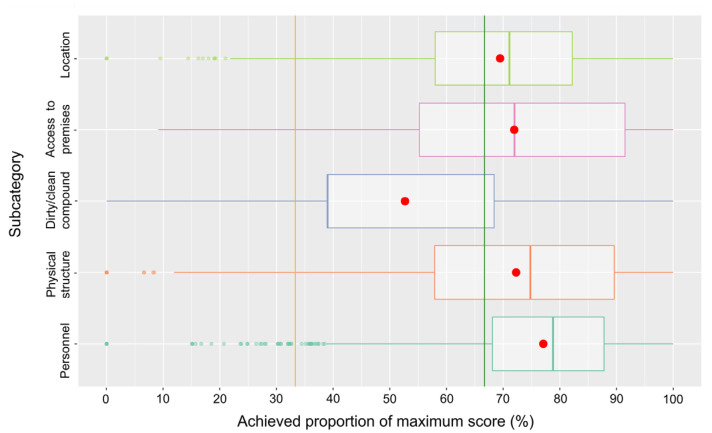
Box-and-whisker plot of the scores in the three subcategories of the main category farm features. The means are indicated as red dots and the risk class thresholds as lines (orange and green).

The results for the subcategories of the main category farm features are presented in Figure 8. While the majority of farms achieved scores qualifying for the low-risk class in the subcategories of location, access to premises, physical structure, and personnel, the median for the subcategory of separation of dirty/clean areas on the compound was markedly below the 66.7% threshold. For the shares of farms falling into the different risk classes, see Table 1.

**Figure 9:**
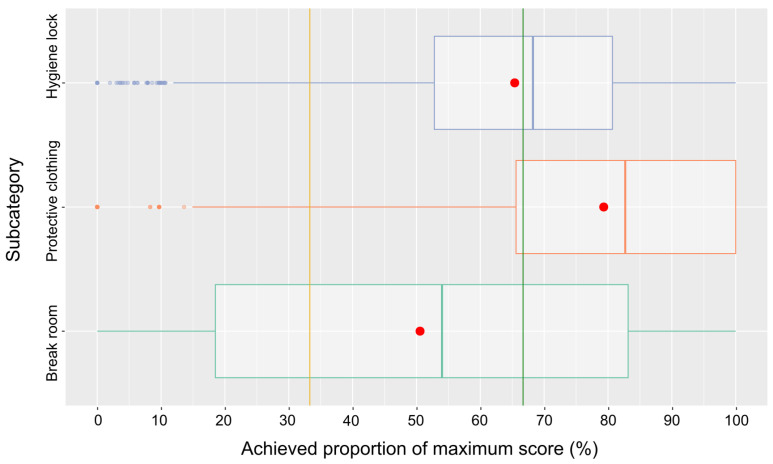
Box-and-whisker plot of the scores in the three subcategories of the main category compound/stable interface. Means are indicated as red dots and risk class thresholds as lines (orange and green).

With regard to the main category compound/stable interface, the results were best for the subcategory protective clothing and poorest for the subcategory break room. The achieved proportion of the maximum score for the subcategory hygiene lock was moderate, with the median slightly above the threshold for the low-risk class (Figure 9, Table 1).

**Figure 10:**
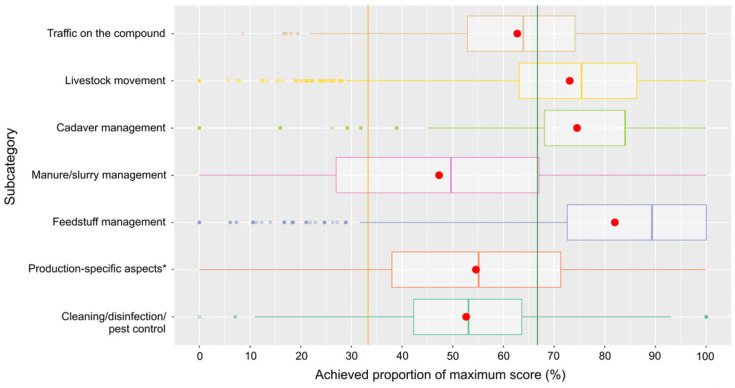
Box-and-whisker plot of the scores in the seven subcategories of the main category management. Means are indicated as red dots and risk class thresholds as lines (orange and green). * Aspects related specifically to fattening, breeding, or mixed production.

Among the subcategories of the main category management, farms performed poorest in the subcategories manure/slurry management, cleaning/disinfection/pest control, production-specific aspects (aspects related specifically to fattening, breeding (piglet production) or mixed production; e.g., all-in/all-out principle for fattening, quarantine for boars concerning breeding, etc.), and traffic on the compound, with the majority of farms achieving results below the threshold for a low-risk classification. On the contrary, for the remaining subcategories of livestock movement, cadaver management, and feedstuff management, most farms ended up in the low-risk class (Figure 10, Table 1).

**Figure 11:**
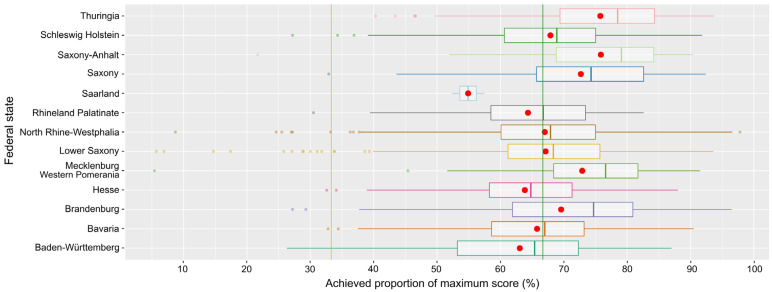
Box-and-whisker plot of the overall scores for the different federal states of Germany; city states with no pig holdings were excluded. Means are indicated as red dots and risk class thresholds as lines (orange and green).

Figure 11 illustrates the overall scores detailed for the different federal states. The distributions differed significantly (Kruskal–Wallis chi-square = 194.78, df = 12, *p*-value < 2.2 × 10^−16^). The federal states in the east of Germany, Thuringia, Saxony-Anhalt, Saxony, Mecklenburg Western Pomerania, and Brandenburg, showed the highest medians, all well above the threshold for the low-risk class. The median for the federal state Saarland in the southwest of Germany was lowest, but still in the upper intermediate class.

**Figure 12:**
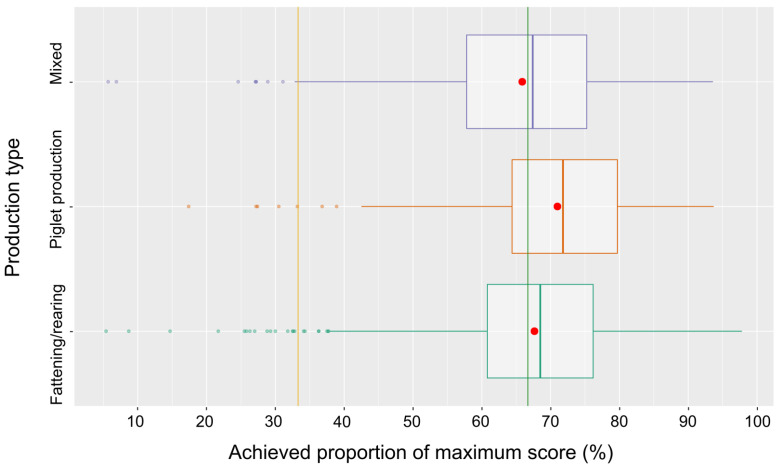
Box-and-whisker plot of the overall scores for the three different production types. Means are indicated as red dots and risk class thresholds as lines (orange and green).

In Figure 12, the overall scores detailed for the different production types are shown. The distributions differed significantly (Kruskal–Wallis chi-square = 43.10, df = 2, *p*-value < 4.5 × 10^−10^). The majority of the farms of all production types achieved scores above the 66.7% threshold for low risk, with the piglet-producing farms (breeding) performing best, followed by fattening/rearing farms and mixed farms.

**Figure 13:**
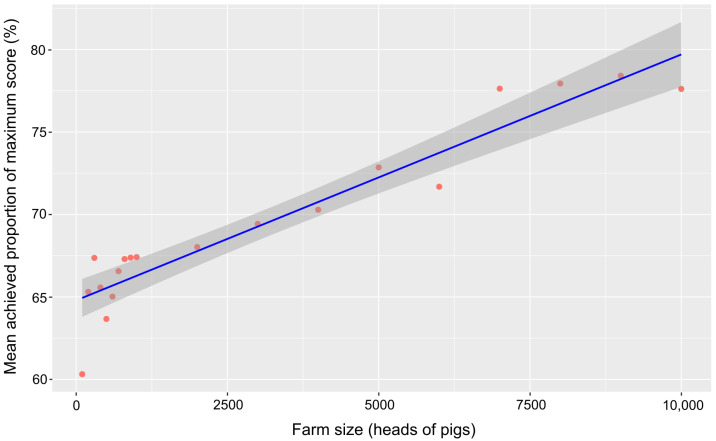
Mean achieved proportion of the maximum score of participating farms in the different selectable farm size classes regressed on the farm size with 95% confidence interval.

There was a significant positive correlation between the mean proportion of achieved scores in different farm size classes, which have to be selected for the farm under evaluation when the tool is used as value from a drop-down list indicating the farm size the true size is closest to (see above), and the farm size class (Figure 13, adjusted R-squared: 0.89, *p* < 0.001).

## 4. Discussion

ASF has been on the rise in Europe since 2007. So far, elimination following entry into the wild boar population has only been successful in the Czech Republic, Belgium, and Sweden. The problem for Germany, as for the neighboring Eastern European countries, is that there is a permanent infection pressure on a broad front, particularly at the eastern borders. Despite the success of disease control, Germany has not been able to eliminate ASF in wild boar in the east of the country on the border with Poland since the first cases occurred in 2020. In 2024, an additional focal area in the west of the country was infected, with many cases of ASF detected in wild boar, and subsequent outbreaks in domestic pigs. In any case, the experience in Europe shows that it is sometimes necessary to live with the threat of ASF in wild boar and possibly also in domestic pigs for many years, which is why shielding farms with effective biosecurity is of the utmost importance.

Though there was already a biosecurity assessment tool available from the University of Ghent (Biocheck.UGent, [19]), it was believed that a tool developed by German experts, incorporating not only European but also national legal requirements of biosecurity and offered in German for German-speaking farmers, would foster applicability and acceptance. The use of an easy-to-understand traffic light scheme was expected to enhance user-friendliness, while tailored suggestions based on the results would further improve the tool’s effectiveness. The risk factors evaluated by the tool covered aspects with a wide range of relevance for ASF incursion, as reflected by the allocated weights. The latter were retrieved from experts in a Delphi process, which considerably consolidated the estimates among the experts. For 107 out of 117 questions, the consistency of the weights was improved.

Between 2019, when the tool was launched, and 2023, the tool was used 3423 times with 2290 complete datasets being fit for analysis. Thus, the application clearly outperformed the use of Biocheck.UGent (228 surveys from Germany as of October, 2024). This is probably, on the one hand, due to language barriers (Biocheck.UGent is available in different languages but not in German) and, on the other hand, the promotion of the ASF traffic light in Germany. Interestingly, the mean overall performance of the abovementioned surveys of German farms with Biocheck (60%) resembled the mean of the farms assessed with the ASF traffic light (67.8%). The frequency of initial utilization was highest in 2019, when the tool was launched, and 2020, when ASF has reached German territory. Since then, the frequency of initial utilization has dropped markedly toward 2023. Those farmers keen to use the tool to optimize biosecurity will have done so already in the previous years. They have received individual to-do-lists and obviously did not feel the need to re-assess their status after the implementation of improvements, which may explain the generally low frequency of second or multiple utilizations of the tool throughout the evaluated years. The provision of customized to-do-lists (specific deficits of the individual farm ranked according to relevance, together with proposed countermeasures) is a very important feature of the tool, especially since a recent study has shown that the awareness of German farmers of dangerous pathways of animal disease introduction into a farm is associated with a lack of knowledge of how to improve the measures in these areas [20]. The overall proportion of utilization in Germany was 11.9%, with marked differences between the federal states. There is a trend, though not significant, for federal states in the east of Germany, located closer to the epidemic of ASF in wild boar, to achieve higher proportions of utilization. It seems to be likely that farmers close to the epidemic are more in favor of critically questioning their own biosecurity standards. But, this impression might be confounded by the farm size. The average farm size of the eastern federal states is much higher than in the west (Table 2 [15], data from May 2022).

This is still due to the structural differences between former West and East Germany, with a higher concentration of pig production in the latter. And, a larger farm size will trigger interest in an optimization of biosecurity via the online tool. Large farms will generally suffer more severe financial losses if affected by disease and pay extra attention to biosecurity. Additionally, larger farms will more easily cope with financially or organizationally challenging measures of biosecurity. In combination, large farms will be more likely to achieve higher biosecurity standards. The latter is supported by the significant positive correlation of the farm size and achieved scores in the ASF traffic light. And, since, as mentioned above, the average farm size is much larger in the eastern federal states, the average achieved scores are higher for the latter as well. The federal states Schleswig Holstein, Lower-Saxony and North Rhine-Westphalia are those federal states of the former West Germany with the largest average farm size and are doing well in the assessment. A recent study specifically in Lower Saxony also found a high level of biosecurity against ASF on most pig farms [21].

In general, the majority of all farms, irrespective of the localization, rank within the “green” range of the scale (median above the 66.7% threshold), indicative of a good biosecurity standard/low risk. The lowest median was found for the federal state Saarland in the very west of Germany, a tiny state with four pig holdings only, of which only two used the tool; so, the results are based on a very small sample and the farm size is lowest in this federal state.

As far as the production type is considered, there are significant differences with respect to the overall biosecurity scores. The breeding farms (piglet producers) achieve the highest scores (mean and median), which is probably due to the higher value of the animals as compared fattening. Farms with mixed production are performing poorest, though the median is still above the low-risk threshold. Mixed production might be more challenging as far as biosecurity is concerned as compared to specialized production.

Although, according to the data, the overall biosecurity level concerning ASFV of the participating farms is considered as good, it remains unclear whether this finding may be extrapolated to all pig holdings in Germany. The results could be biased toward higher scores if farms with a generally higher awareness of biosecurity were more likely to take the online assessment. Those farms will probably already have established relatively high standards. On the other hand, farms suspecting deficiencies in biosecurity could also be attracted, potentially levelling out the aforementioned bias. At least, because it guarantees anonymity and offers an individual to-do-list for improvement, the tool intends to attract farms irrespective of their presumed biosecurity standard.

The distributions of scores for the three main categories of farm features, compound/stable interface, and management are only principally comparable with medians well in the low-risk range for farm features and compound/stable interface, but management is falling behind. Among the subcategories of the main category farm features, most of the farms scored very high for biosecurity aspects related to the control of access to the premises, the physical structure of the farm and its buildings, and aspects of the personnel operating the farm, categories that may more readily be influenced by the farm operator than aspects of farm location, e.g., the proximity of other pig holdings. Consequently, the scores for the latter subcategory are lower, though only slightly. The subcategory with aspects related to a separation of clean and dirty areas on the compound yields quite poor results, with a median not much above the 33.3% threshold of a high risk. It is obviously more difficult to ensure such a separation on the compound (e.g., avoiding the crossing of routes of personnel, vehicles, etc.) than at structurally more defined interfaces like access to the premises (e.g., fencing) and the integrity of buildings. Among the subcategories of the main category compound/stable interface, performance concerning the break room was poorest, because many farms do not provide such a room at all. Scores for protective clothing were very high. Obviously, this aspect is perceived as very important to avoid biosecurity breaches. Unfortunately, scores for the hygiene lock were also relatively high but markedly fell behind those for protective clothing. It could be speculated that establishing a proper physical hygiene lock is more demanding than just changing clothes/shoes and related aspects such as keeping clean and dirty clothes apart. Focusing on the subcategories of the last main category, management, most subcategories yielded very high or acceptable (traffic on compound) scores, with the exception of manure/slurry management, production-specific aspects, and cleaning/disinfection/pest control. For manure/slurry management, many questions were not answered and most farms stocked manure and slurry on the compound where the stables are located. Concerning the production-specific aspects, many answers were missing as well, or the unfavorable answers were ticked. Special attention should be paid to the poor results for cleaning/disinfection/pest control. Only 15.5% of the farms achieved scores in the low-risk range. Many farms did not have a cleaning and disinfection plan, indicated insufficient rodent control as well as access control for dogs and cats, and did not carry out disinfection of vehicles.

The biosecurity assessment tool “ASF traffic light” is quite well accepted but has further potential concerning re-assessments of farms and assessments of farms that did not use the tool yet or are about to go into business. The reason for the reduction in utilization until 2023 might be, on the one hand, due to the perception that ASF was basically constrained to the very east of Germany since its introduction 2020. On the other hand, it is known that on many farms, the downloadable questionnaire of the tool is used to assess biosecurity offline. But, the latest data from 2024 (initial utilization, 670; second or multiple utilizations, 41) demonstrate that the frequency of use has increased markedly again. The numbers are resembling those of 2020, which might be due to the startling establishment of a new cluster of disease (wild boar and domestic pig) in the west of Germany in mid-2024. The potential of the tool to satisfy certain requirements of EC legislation with respect to farm-specific risk assessments, the provision of biosecurity plans, and training will likely also lead to a wider use. A recent survey [21] with respect to ASF found a realistic perception of farmers regarding their own biosecurity, which indicates that relatively unbiased self-assessments, which the ASF traffic light is depending on, are possible. Participating farmers are, of course, informed in the preamble of the tool that only realistic inputs will generate a reliable assessment and helpful recommendations for improvement. The evaluation of the results of farms that participated so far offers valuable insights into the status quo of German pig farming with respect to biosecurity and allows to specifically address identified frequent weaknesses in official controls, trainings, presentations, etc. Especially with regard to cleaning and disinfection, pest control, the implementation of an efficient hygiene lock, the separation of clean and dirty areas on the compound, and the traffic on the latter, there is room for improvement. In the light of the threats pathogens pose to pig farming and the value chain, particularly ASF at present, raising the level of biosecurity will be worth the effort to strengthen the resilience of the sector.

## Figures and Tables

**Table 1:** Measures of the central tendency of biosecurity scores and proportion of farms in the three risk classes for the different categories (n = 2293).

Main Category	Subcategory			Percentage in Risk Class		
		Mean	Median	Low	Intermediate	High
**All (overall score)**		67.8	68.8	1.3	41.1	57.6
**Farm features**		71.1	71.8	0.7	33.8	65.5
Location		69.5	71.1	2.0	38.1	59.9
Access to premises		71.9	72.0	4.8	34.2	61.0
Clean/dirty separation on compound		52.7	39.0	11.9	47.8	40.3
Physical structure		72.3	74.8	3.7	31.4	64.9
Personnel		77.1	78.8	1.4	21.0	77.6
**Compound/stable interface**		66.8	68.9	7.0	38.4	54.6
Hygiene Lock		65.4	68.3	8.7	37.8	53.5
Protective clothing		79.3	82.7	5.1	21.0	73.9
Break room		50.6	54.0	29.4	32.9	37.7
**Management**		65.3	66.4	1.5	49.6	48.9
Traffic on the compound		62.7	63.9	2.8	55.8	41.4
Livestock movement		73.1	75.4	3.1	30.0	66.9
Cadaver management		74.5	84.0	4.5	20.3	75.2
Manure/slurry management		47.3	49.7	31.1	42.9	26.0
Feedstuff management		82.0	89.3	5.6	13.4	81.0
Production-specific aspects ^1^		54.6	55.1	13.9	59.0	27.1
Cleaning/disinfection/pest control		52.6	53.1	8.8	76.0	15.2

^1^ Aspects related specifically to fattening, breeding, or mixed production.

**Table 2:** Mean farm size (heads of pigs) in the German federal states without city states.

Federal State	Mean Farm Size
Saxony-Anhalt ^1^	5841
Thuringia ^1^	4596
Mecklenburg Western Pomerania ^1^	4583
Brandenburg ^1^	4509
Saxony ^1^	3876
Schleswig Holstein	1889
Lower Saxony ^1^	1665
North Rhine-Westphalia	1010
Baden-Württemberg	783
Rhineland Palatinate	726
Bavaria	704
Hesse	631
Saarland	170

^1^ Federal state located in the former East Germany.

## Data Availability

The data are available in the manuscript and Supplementary Materials.

## Supplementary Materials

The following supporting information can be downloaded at https://www.mdpi.com/article/10.3390/pathogens14060524/s1 Table S1. Questionnaire, Table S2. Farm assessment data, Table S3. Glossary, Table S4. Overview.
